# Detection of Falsified Antimalarial Sulfadoxine-Pyrimethamine and Dihydroartemisinin-Piperaquine Drugs Using a Low-Cost Handheld Near-Infrared Spectrometer

**DOI:** 10.1155/2022/5335936

**Published:** 2022-05-03

**Authors:** Moussa Yabré, Abdoul Karim Sakira, Moumouni Bandé, Bertrand W. F. Goumbri, Sandrine M. Ouattara, Souleymane Fofana, Touridomon Issa Somé

**Affiliations:** ^1^Higher Institute of Health Sciences (INSSA), Nazi BONI University, Bobo-Dioulasso, 01 P.O. Box 1091, Burkina Faso; ^2^Laboratoire de Toxicologie Environnement et Santé (LATES), Joseph KI-ZERBO University, Ouagadougou, 03 P.O. Box 7021, Burkina Faso

## Abstract

Falsified drugs are of serious concern to public health worldwide, particularly for developing countries where quality control of drugs is inefficient. In law enforcement against such fake medicines, there is a need to develop reliable, fast, and inexpensive screening methods. In this work, the ability of an innovative low-cost handheld near-infrared spectrometer to identify falsifications among two antimalarial fixed dose combination tablets, dihydroartemisinin/piperaquine and sulfadoxine/pyrimethamine, has been investigated. Analyzed samples were collected in Burkina Faso mainly in rural transborder areas that could be infiltrated by illicit drugs. A principal component analysis was applied on the acquired near-infrared spectra to identify trends, similarities, and differences between collected samples. This allowed to detect some samples of dihydroartemisinin/piperaquine and sulfadoxine/pyrimethamine which seemed to be falsified. These suspicious samples were semiquantitatively analyzed by thin-layer chromatography using Minalab® kits. Obtained results allowed to confirm the falsifications since the suspected samples did not contain any of the expected active pharmaceutical ingredients. The capacity of the low-cost near-infrared device to identify specifically a brand name of dihydroartemisinin/piperaquine or sulfadoxine/pyrimethamine has been also studied using soft independent modelling of class analogy (SIMCA) in the classical and data driven versions. The built models allowed a clear brand identification with 100% of both sensitivity and specificity in the studied cases. All these results demonstrate the potential of these low-cost near-infrared spectrometers to be used as first line screening tools, particularly in resource limited laboratories, for the detection of falsified antimalarial drugs.

## 1. Introduction

According to the World Health Organization (WHO), falsified medicines are defined as products that deliberately or fraudulently misrepresent their identity, composition, or source [1]. Fake medicines are of serious concerns to public health worldwide, particularly for developing countries where regulatory systems are weak and quality control of drugs is inefficient. The impacts of fake medicines include treatment failure, antimicrobial resistance, morbidity, and mortality increase [2]. It has been estimated that approximately 10% of medicines administered worldwide are of poor quality [3–5]. Even if all pharmacological classes are affected, vital drugs like antimalarials remain ones of the most falsified, particularly in developing countries [6]. Indeed, it has been estimated that 35% of antimalarial medicines in Sub-Saharan Africa failed chemical analysis, and 20% were falsified [7]. The consumption of such poor quality antimalarial drug may be associated annually to 120000 deaths of under-five children [8].

Drug quality control is a key issue in the supply chain monitoring, law enforcement, and ensuring patient protection [9]. It is generally performed according to pharmacopeias which involved analytical techniques such as liquid or gas chromatography. These techniques are expensive to perform, particularly for resource limited laboratories. In order to bridge the capacity gap of drug quality monitoring of resource limited countries, the Global Pharma Health Fund (GPHF) developed field test kits, called Minilab^®^, based on semiquantitative thin-layer chromatography allowing the detection of falsified and grossly substandard drugs [8, 10, 11]. Although Minilab^®^ kit is less expensive and easy to implement, it is destructive, requires sample preparation steps, and uses reagents which are sometimes harmful and of environmental concerns.

Near-infrared spectroscopy is an analytical technique well known for its potential in the detection of falsified medicines [12, 13]. It has also the advantages of being nondestructive, fast, requiring little or no sample preparation steps, as well as being environmentally friendly. The bands in NIR predominantly arise from overtones and combination of stretching of O–H, C–H, and N–H bonds are generally much broader and weak than those seen in the middle infrared region, therefore giving a lower molecular selectivity [14]. That is why near-infrared analysis is generally combined with chemometrics [15, 16]. Near infrared spectroscopy associated to chemometrics is more and more employed for product identification and particularly detection of falsified drugs [2, 16, 17]. However, the high cost of classically commercialized instruments limit their use, particularly in resource limited laboratories.

Recently, some innovative handheld and low-cost NIR spectrophotometers have been commercialized. These low-cost devices are very compact and can operate autonomously using batteries [18]. They are also provided with wired USB and bluetooth wireless connections that made them compatible with microcomputers, tablets, or cell phones. Besides their low cost, they can offer promising performance comparable to bench-top instruments [19, 20]. Their potential has been assessed in the detection of falsified antimalarial artemether and lumefantrine drugs [9, 19].

We report in this study the ability of a low-cost NIR spectrometer as a screening tool to identify falsifications among two fixed dose antimalarial combination tablets, dihydroartemisinin/piperaquine (DP), and sulfadoxine/pyrimethamine (SP). Samples from different brands were collected in Burkina Faso mainly in rural transborder areas that could be infiltrated by medicines trafficking. A principal component analysis (PCA) was first applied as an exploratory tool on the acquired spectra to identify trends, similarities, and differences between collected samples and detect suspicious falsified samples. The suspicious samples were then analyzed using Minalab^®^ kits to confirm falsifications. The potential of the NIR spectrometer to identify specifically a brand name of DP or SP using soft independent modelling of class analogy (SIMCA) in the classical and data driven versions has been also investigated.

## 2. Materials and Methods

### 2.1. Chemicals and Drug Products

Dihydroartemisinin-piperaquine (DP) and sulfadoxine-pyrimethamine (SP) tablets, both in fixed dose combination, were collected in Burkina Faso in different transborder zones (Table 1). All samples collected were from licit drugstores, except for some Maloxine® samples which were bought with illicit street vendors. Except Duo-Cotecxin® and Maloxine® for which at least 5 batches were sampled, only two batches were collected for each product. In fact, it was difficult to get more batches during the sample collection because the same sample batches were found in the different drugstores at the time of the study.

After the NIR analysis, samples were further semiquantitatively analyzed by thin-layer chromatography (TLC) using the Global Pharma Health Fund (GPHF) MiniLab^®^ kits^®^ and protocols [21]. The reference standards (all in tablet formulation) employed for the TLC analysis were also from GPHF and were kindly provided by the national public health laboratory of Burkina Faso.

### 2.2. Near-Infrared Analysis

#### 2.2.1. Instrumentation

Analyzes were performed using NIR-S-G1 spectrophotometer from InnoSpectra (Hsinchu, Taiwan). It is a low-cost (less than 1000 €) handheld dispersive near-infrared instrument which can operate autonomously using batteries [22]. The NIR-S-G1 spectrophotometer can be driven by computers, tablets, or cell phones using wired USB and bluetooth wireless connections. It allows to monitor the 900–1700 nm near-infrared spectral region with a nominal resolution of 10 nm.

#### 2.2.2. Data Acquisition

Tablet samples were directly scanned through their transparent blister, except for Maloxine® and Fansidar® samples. For these latter, spectra were directly recorded on the bare tablets because of the opacity of their primary packaging. Spectra of ten tablets per batch were recorded in the 900–1700 nm region for each formulation. Therefore, a total of 230 spectra was acquired.

#### 2.2.3. Spectral Preprocessing

Prior to chemometric analysis, appropriate pretreatments of the acquired near-infrared spectra were necessary to eliminate irrelevant information which are mainly due to differences in physical characteristics of the samples. Therefore, data preprocessing was used to improve signal-to-noise ratio. The preprocessing consisted of a Savitzky-Golay smoothing and differentiation filter (second-degree polynomial and second derivative) followed by a multiplicative scatter correction (MSC). The chemometric analysis was performed on the spectral range between 1085 nm and 1601 nm because the other spectral areas were found noisy and less repeatable.

#### 2.2.4. Principal Component Analysis

Principal component analysis (PCA) is a common unsupervised technique which forms the basis for multivariate data analysis [16]. It allows the exploration of data through the reduction of its dimensionality [23]. In fact, PCA allows to reduce the dimensions of the original data space by using a smaller and more efficient abstract space of latent variables called principal components (PCs) [24]. In this new space, data (spectra in our case) can be displayed while keeping the same information as the original space. Each spectrum is visualized as a point in a two or three dimensional plot defined by the selected principal components (PCs). Usually, the first three principal components are the most informative and explain the variance in the data. PCA allows to enhance similarities and differences between the spectra, allowing the detection of underlying clusters.

#### 2.2.5. Soft Independent Modelling of Class Analogy (SIMCA)

A classification method was built to evaluate the ability of the low-cost instrument to authenticate specifically a drug brand name. Chemometric models based on a class modelling or one-class classifier like soft independent modelling of class analogy (SIMCA) are more recommended for authentication purpose [25, 26]. The original version of SIMCA has several modifications mostly related to the way of building the acceptance boundaries. A recent known modification is data driven (DD)-SIMCA [27]. Both original and data driven versions of SIMCA were used in this study.

*(1) Classical SIMCA Analysis*. The classical SIMCA algorithm uses samples with known origin (training samples) to perform a classification rule which allows classifying new samples (test samples) in one of the classes [24]. The different classes are modelled individually by a separate PCA. The number of PCs was chosen for each class using a venetian blinds cross-validation. PCA results are then used to estimate the residual *Q* and the Hotelling T2 statistics from the calibration data. The classification of a sample is based on the *Q* and T2 for the sample and the estimation of the T2 and *Q* distributions from the training data. This allowed to compute confidence limits set at 95%. With the PLS Toolbox software, these confidence limits are used to calculate the probability of a sample to be in a given class. A sample is attributed to a class if the probability is greater than a specified threshold probability value fixed at 0, 8 in this work. SIMCA models were built for each product. For Duo-Cotecxin® and Maloxine® products for which at least five batches have been collected, spectra of three batches were used as a training set for the model building and the two remaining batches were used as a test set. For the products for which only two batches were collected, Kennard-Stone algorithm was used to split the collected spectra into training (60 percent of spectra) and test (remaining 40 percent of spectra) sets for each product. Falsified samples were integrated only to the test set.

*(2) DD-SIMCA Analysis*. SIMCA in its data driven version was also used to build a classification model for 2 target classes: Duo-Cotecxin® and Maloxine® products for which falsified samples have been identified and enough batches were collected. Like any SIMCA model, DD-SIMCA decomposes the training spectra of the target class by PCA [19, 26, 27]. Then, the results of PCA decomposition are used to compute a score distance (*hi*) and an orthogonal distance (*vi*) for each training sample [25, 28]. Each type of distance is modelled using a scaled chi-squared distribution instead of the residual *Q* or Hotelling T2 statistics used in the original SIMCA models [24]. The calculated score and orthogonal distances are used to define the acceptance area or thresholds for the target class at a given significance level *α*. The DD-SIMCA models are usually shown using a two-dimensional plot with a limit curve allowing to determine whether or not the samples belong to the target class [29]. For each Duo-Cotecxin® and Maloxine® product, spectra of three batches were used as a training set to build the model and the two remaining batches were used as a test set to evaluate the model sensitivity. The other DP and SP products were employed to mimic high quality fake drugs and test model specificity.

The performance of the classical and data driven versions of SIMCA modelling was assessed based on sensitivity and specificity. Sensitivity is related to the percentage of samples from the target class that are correctly attributed as a member of the target class. Specificity is related to the percentage of samples from nonmembers of the target class, which are properly attributed as nonmembers of the target class [29].

### 2.3. Software

The spectral preprocessing, the PCA, and the classical SIMCA modelling were carried out using the PLS_Toolbox version 8.9.2, and while the DD-SIMCA analysis was done using DDSGUI, a graphical user interface freely available online [30]. All chemometric procedures were performed in a MATLAB environment (R2019a).

## 3. Results and Discussion

### 3.1. Spectral Data Pretreatment

**Figure 1:**
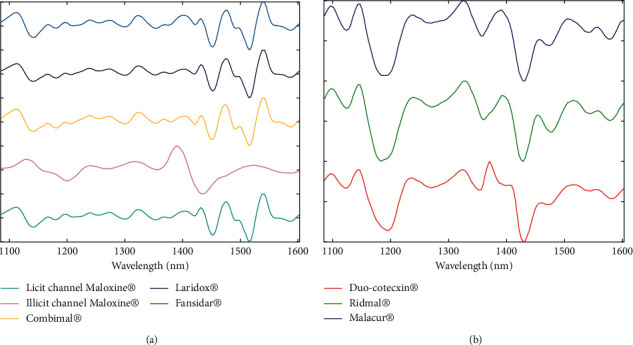
Preprocessed mean spectra in the 1085–1601 nm range. (a) SP samples. (b) DP samples.

Figure 1 illustrates the pretreated selected spectral range prior the modelling process. The second derivative was chosen to remove noise and baseline signals. The multiplicative scatter correction (MSC) was then applied to the smoothed and differentiated signals.

### 3.2. Principal Component Analysis (PCA)

**Figure 2:**
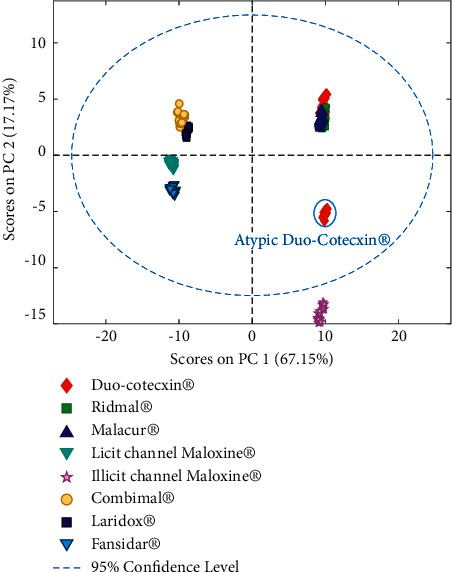
PC1-PC2 score plot of the preprocessed data of both SP and DP data.

**Figure 3:**
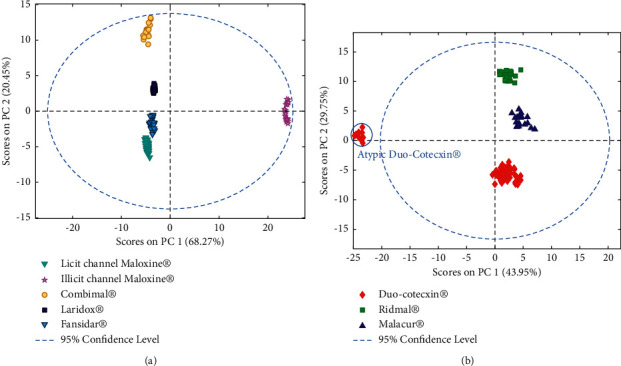
PCA applied separately on SP and DP preprocessed data. (a) SP PC1-PC2 score plot. (b) DP PC1-PC2 score plot.

A PCA was carried out on the acquired and pretreated spectra to enhance differences and similarities between the spectra and to identify underlying clusters [17]. The PCA was first carried out simultaneously on DP and SP samples.  Figure 2 presents the score plot for the spectra of both DP and SP samples in the space spanned by the first (PC1) and second (PC2) principal component. These two PC explained nearly 85% of the variability. In a second time, PCA was applied separately on SP and DP samples (Figure 3).

#### 3.2.1. SP Product Analysis

The PC1-PC2 score plot, presented in Figure 2, allowed to notice that the spectra of all SP products from the licit sale channel were grouped together and could be distinguished from DP spectra. Also, one can see that the samples of the illicit channel Maloxine® were far from the samples of the licit channel Maloxine® and other SP products, being outside the 95% confidence level. The PCA applied only on SP products allowed to confirm that SP samples from licit sale channel were similar but very different from the illicit channel samples of Maloxine® (Figure 3(a)). Therefore, these samples bought from illicit street vendors seemed to be falsified.

#### 3.2.2. DP Product Analysis

The PCA applied on both SP and DP products allowed to see that DP spectra were also grouped, except some spectra of Duo-Cotecxin® which were far from other spectra of Duo-Cotecxin® and other DP medicines (Figure 2). These isolated Duo-Cotecxin® spectra were all from the same batch. Therefore, this batch appeared to be very different from the 5 other batches of Duo-Cotecxin®. For a better visualization, a PCA was also applied only on DP products (Figure 3(b)). This allowed to confirm the atypic behavior of one sample of Duo-Cotecxin® since its scores were very different from other Duo-Cotecxin® samples and DP formulations. Therefore, this sample appeared to be also falsified as the samples of the illicit channel Maloxine®.

The PCA applied separately on SP and DP products (Figure 3) allowed to differentiate each DP formulations on the one hand and each SP product on the second hand even if all these formulations seemed to contain the correct expected active pharmaceutical ingredients. This can be explained by the fact that the analyzed medicines may not have the same nature and composition of excipients and that NIR spectra are sensitive not only to chemical properties but also to physical properties.

### 3.3. SP and DP Sample Analysis Using MiniLab® Kit

To confirm NIR results, all SP and DP tablets were analyzed using MiniLab^®^ kits which allow a rapid drug quality verification through a semiquantitative thin-layer chromatography [21]. MiniLab^®^ kits are reliable to detect grossly substandard (less than 80% of the expected amount) or wrong drug samples [8, 11]. As expected from the NIR analysis, none of the expected active pharmaceutical ingredient was found in samples of Maloxine® from the illicit sale channel (neither sulfadoxine, nor pyrimethamine and in the suspected Duo-Cotecxin® sample (neither dihydroartemisinin, nor piperaquine). On the contrary, all other Duo-Cotecxin® samples and DP and SP products passed with success in Minilab^®^ tests and appeared to contain at least 80% of the expected amount of the respective active pharmaceutical ingredients.

The falsification of Maloxine® samples was expected since they were purchased with illicit street vendor and their packaging was different of the packaging of licit Maloxine® samples. On the contrary, the falsified Duo-Cotecxin® sample was purchased in a licit drugstore and the visual inspection did not allow to notice a significant difference in comparison with the other Duo-Cotecxin® samples. These results allowed to demonstrate the great potential of the low-cost NIR spectrometer as a screening tool for the detection of falsified drugs without API.

### 3.4. SIMCA Analysis

Considering PCA results, a classification method was investigated to evaluate the potential of the low-cost instrument to authenticate specifically a given brand name of SP and DP formulation. A class modelling method like SIMCA which is recommended for the verification of the identity of products [19, 26] has been used both in its classical version and data driven one.

#### 3.4.1. Classical SIMCA Analysis

Original SIMCA models were constructed for each DP and SP product. Six SIMCA models were built and a correct classification rate of 100% of both sensitivity and specificity was obtained (Table 2). Falsified samples were assigned to no built class. The created SIMCA models allowed to differentiate each SP and DP product from other formulations even if all these formulations contain the correct expected active pharmaceutical ingredients. This can be explained by the fact that the analyzed drugs may not have the same nature and composition of excipients and that NIR spectra are sensitive to both chemical and physical properties.

#### 3.4.2. DD-SIMCA Analysis

**Figure 4:**
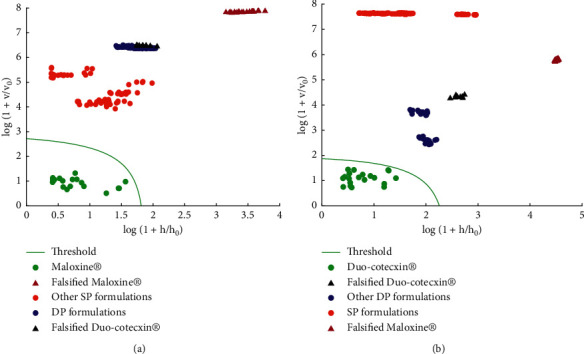
Data driven soft independent modelling of class analogy (DD-SIMCA) plots. (a) Maloxine® (two PCs, *α* = 10^−6^). (b) Duo-Cotecxin® (two PCs, *α* = 10^−7^).

DD-SIMCA models were constructed only for Duo-Cotecxin® and Maloxine® formulations for which falsified samples have been identified and enough batches have been collected. Results of built DD-SIMCA models for the two target classes are shown in Figure 4. The DD-SIMCA models allowed, like classical SIMCA, a clear product authentication and thus a specific brand identification with 100% of both sensitivity and specificity for the studied cases.

These results showed that despite the limited spectral range and low resolution of this low-cost spectrophotometer, it offers promising performance as a screening tool for proper falsification detection and specific brand identification of the antimalarial dihydroartemisinin/piperaquine and sulfadoxine/pyrimethamine drugs.

## 4. Conclusion

The obtained results allow affirming that these innovative low-cost portable near-infrared spectrometers, associated to chemometric tools, offer promising performance to be used as an analytical method for routine testing against pharmaceutical falsification of antimalarial dihydroartemisinin/piperaquine and sulfadoxine/pyrimethamine drugs in their intact form. Despite their limited spectral range and low resolution, these devices allowed detecting falsified drugs with no active pharmaceutical ingredient and identifying specifically a brand name. This innovative handheld NIR spectrophotometer could be used as a first line screening tool in the detection and fight against antimalarial falsified drugs, particularly in developing countries. The implementation of such screening devices combined to a better monitoring of the medicine supply chain would reduce the infiltration of falsified drugs in licit drugstores.

## Figures and Tables

**Table 1:** DP and SP collected tablets.

Brand name	API	Dosage (mg)	Sales channel	Tested batches
Duo-Cotecxin®	DP	40–340	Licit drugstore	6
Ridmal®	DP	40–340	Licit drugstore	2
Malacur®	DP	40–340	Licit drugstore	2
Maloxine®	SP	500–25	Licit drugstore	5
Maloxine®	SP	500–25	Illicit street vendors	2
Combimal®	SP	500–25	Licit drugstore	2
Laridox®	SP	500–25	Licit drugstore	2
Fansidar®	SP	500–25	Licit drugstore	2

API: active pharmaceutical ingredient.

**Table 2:** classical SIMCA model parameters.

Class	Number of PC	Sensibility (%)	Specificity (%)
Duo-Cotecxin®	6	100	100
Ridmal®	3	100	100
Malacur®	3	100	100
Maloxine®	3	100	100
Combimal®	2	100	100
Laridox®	2	100	100

## Data Availability

The (mentioned or referenced) data used to support the findings of this study are included within the article.
